# Attitude and Needs Toward MTM Applications of Chronic Disease in China: A Questionnaire Survey

**DOI:** 10.3389/fpubh.2022.812709

**Published:** 2022-07-29

**Authors:** Shiqiong Huang, Juanjuan Huang, Xuanyu Deng, Lihui Ouyang, Gefei He, Ji Sun

**Affiliations:** Department of Pharmacy, The First Hospital of Changsha, Changsha, China

**Keywords:** attitude and needs, medication therapy management, chronic disease, WeChat applet, questionnaire survey

## Abstract

**Objective:**

Chronic diseases are characterized by high incidence, long-term medication, and complex types of medication. There are also many corresponding medication therapy management (MTM) applications on the market, such as iCarea, and Medisafe. However, the existing research mainly focuses on how to choose high-quality MTM applications, and few researchers consider the expectations of MTM applications from potential users. The aims of this study were to investigate the demand, attitude, and expectations of the Chinese patients for the MTM applications to support.

**Methods:**

From August 2019 to December 2019, we created a questionnaire to have knowledge of user needs, preferences, and expectations for MTM applications among 302 chronic patients in Hunan, Guangdong, and other provinces in China. Logistic regression analysis was performed to analyze the risk factors of affecting patients' attitudes toward MTM applications. Then, respondents' expectations and preferences for MTM applications were statistically analyzed. The survey data were merged to provide information for the design of targeted chronic disease MTM applications.

**Results:**

A total of 260 (86.09%) out of 302 patients the respondents were willing to use the MTM applications of chronic disease. The independent influencing factors for using the MTM applications were long-term medication history (OR = 4.45, *P* < 0.001), willing to learn about medicine knowledge (OR = 3.01, *P* = 0.04), and wanting to get more professional medication knowledge *via* Internet (OR = 2.86, *P* = 0.005). It was worth noting that among those willing to use MTM applications, 55.00% of respondents were willing to use the WeChat applet for MTM, while only 23.46% of respondents preferred other applications. As to the more prevalent WeChat applet for MTM, the majority of participants expected the inclusion of useful modules, such as medication log (62.81%), medication reminder (62.81%), and medication recommendations (57.79%).

**Conclusion:**

The participants are willing to use MTM applications of chronic disease, with a preference for the WeChat applet. Patients tended to use MTM applications if they had a long-term medication history or a desire for medical knowledge, especially if they want to get more professional medication knowledge *via* the Internet. Participants are expected to include in the WeChat applet as medication logs, medication reminders, and medication recommendations which should be taken into serious account for the further development of MTM applications.

## Introduction

According to a systematic analysis for the Global Burden of Disease Study 2015, 71% of deaths worldwide are attributed to chronic diseases, mainly including cardiovascular disease, diabetes, and chronic kidney disease (1). It is worth noting that chronic diseases in China in 2019 accounted for 88.5% of the total deaths in the “Report on Nutrition and Chronic Diseases of Chinese Residents” (2). The number of chronic disease patients is increasing, which seriously threatens the health of residents and becomes a major public health issue affecting economic and social development (3). Characterized by high morbidity, long-term medication, and complex types of medication, chronic diseases lead to medication misunderstanding and poor medication adherence in most patients, which is closely linked to increased morbidity, mortality, and medical costs, making the long-term treatment of chronic diseases a global concern (4–7).

Medication therapy management (MTM) has been defined as a unique service that optimizes dosing regimen by collecting information on the patient's medical and drug history, drug monitoring, and other aspects (7, 8). The MTM services resulted in significant improvements in therapeutic and safety outcomes compared to conventional care, such as lowering patients' blood pressure, Hemoglobin Ale, and low-density lipoprotein, and optimization of the patient's treatment regimen to reduce the risk of adverse events and healthcare costs (9, 10). Consequently, as a professional pharmaceutical means of disease management, the MTM service model can not only effectively guarantee the rational medication of chronic disease patients, but also remarkably reduce the economic burden of the country and patients. However, due to the shortage of medical resources, the lack of in-depth community medical services, the relatively backward medical technology in remote areas, and other reasons, there is much more difficult for the current medical system to provide “one-to-one” pharmaceutical care with the aid of traditional MTM services (11).

Patients of all ages and conditions face challenges when taking medications, such as integrating medications into their daily lives at the right time, understanding medications and their effects and side effects (12), or tracking their symptoms and treatments (13). To achieve these goals, a reliable and easy-to-use medication management tool becomes essential. Thanks to the universality of the Internet and smartphone, online MTM applications are becoming increasingly widely used to achieve these goals (14). Studies have shown that patients with uncontrolled hypertension aided by MTM application (electronic health record) provided by pharmacists can obtain better blood pressure control (15). Using MTM applications (AllyQuest) in HIV-positive young people is associated with better antiretroviral therapy adherence (16). Software developers are aware of this potential to make the mobile application market grow, creating many MTM applications available, such as iCarea and Medisafe (17). These applications can help patients take medication correctly and avoid medication errors, especially for patients requiring complex medications, such as those with cardiovascular diseases (18–21). Nevertheless, there is still limited knowledge on public attitudes and needs toward MTM applications, such as the WeChat applet to support MTM of chronic disease. Therefore, in this study, we designed a questionnaire to survey patients with chronic diseases, aiming to analyze patients' needs and attitudes toward MTM applications and provide some insight into the further development of the MTM application.

## Materials and Methods

### Study Design

#### Overview

To create the required questionnaires, we reviewed a large number of published papers on MTM applications, and created a questionnaire surveying patients' attitudes and needs for MTM in chronic diseases based on the questionnaires used in these published studies.

#### Questionnaire Design

In the initial stage of this research, by consulting scientific materials, including reference books and MTM applications related to chronic diseases, we produced a preliminary draft of the questionnaire containing 28 questionnaire items. The final edition of questionnaire consisted of the following three sections: (i) Basic information about the respondents, including gender, age, education, location of the family, whether or not having multiple chronic diseases, compliance of medication, willingness to learn about medicine, whether or not having long-term medication history (≥3 months), etc. (ii) Smartphone usage: hours per day using the smartphone, whether or not using smartphone as main source of medication knowledge, etc. (iii) The MTM application for chronic disease: intention to using MTM application, the desired form of MTM applications (WeChat applet), the expected future modules of MTM applications, factors impeding the use of MTM applications (excessively professional expression of medical knowledge on MTM application, risk of user information leakage and risk of missing medication reminder), and future expectation of MTM applications (simple and convenient to operate, full of illustrations and a user-friendly interface).

#### Relevance and Clarity

In reference to Shahmoradi's study (22), the questionnaire used in this study were evaluated for content validity by 18 experts in the field of chronic disease treatment, including doctors, nurses, and pharmacists. The items in the initial questionnaire were corrected and clarified according to the content validity index (CVI). The 28 items of the questionnaire were initially selected and distributed to 18 experts to collect their feedback on their relevance to this study and clarity on a scale of 1–4, where 1 indicated no relevance or clarity and 4 indicated high relevance or clarity. If four or more experts rate the relevance of certain items 1 or 2, it should be deleted from the questionnaire. If any expert rates the clarity of the statement no more than 3, the wording of the statement should be adjusted.

Revision and confirmation of the final questionnaire: According to the enlightening results of experts, 5 items were deleted because of ambiguous statements or irrelevance to this study. There were another 5 items whose verbal expression was clarified after we held several face-to-face meetings with those 18 experts. At the end of the discussion, it was determined that the questionnaire included 23 final items.

### Study Population

The questionnaires were collected by using the questionnaire of mobile phones over a period of 5 months, from August to December 2019. The subjects of the study were patients diagnosed with chronic diseases in The First Hospital of Changsha, such as hypertension, coronary heart disease, diabetes, hyperlipidemia, and other chronic diseases. Inclusion criteria: (1) age: from 18 to 90 years; (2) having the ability to independently use mobile phones to fill out questionnaires; (3) no professional medical knowledge (4) agreeing to cooperate with the investigation and signing the informed consent. Exclusion criteria: (1) patients with severe mental illness; (2) disagree to participate in this survey.

### Ethics Approval

This study was approved by the Institutional Ethics Committee of the First Hospital of Changsha and was conducted in accordance with the Declaration of Helsinki. All enrolled participants were provided written informed consent.

### Data Processing and Statistical Analysis

Numbers and percentages were used to represent categorical variables. All analyses were carried out using SPSS, version 25. Statistical significance was analyzed using analysis of variance and *t*-tests as appropriate. Both univariate and multivariate analyses were carried out to measure the association of some potential factors and intention to use MTM applications. Results were presented as percentages and frequencies as appropriate. To test statistical significance, 95% CIs and/or *p*-values were used. A *p*-value < 0.05 was regarded as being statistically significant.

## Results

### The General Characteristics

The types of chronic diseases of participants were shown in Table 1. A total of 302 participants were included in this study. According to respondents' willingness to use MTM applications, they were divided into the “Willing to use MTM applications” group (positive attitude group) and the “Unwilling to use MTM applications” group (negative attitude group), among which 260 (86.09%) belonged to the former and 42 (13.91%) belonged to the latter group. There were no significant differences in disease types between people who intended to use the MTM applications and those who did not.

**Table 1:** Chronic disease type of subjects.

**Disease types**	**Total (%)**	**Willing to use MTM applications**	**Unwilling to use MTM applications**	***P*-value**
	**(*****n** **=*** **302)**	**(*****n** **=*** **260)**	**(*****n** **=*** **42)**	
Hypertension	125 (41.39%)	113 (43.46%)	12 (28.57)	0.069
Diabetes mellitus	27 (8.94%)	24 (9.23%)	3 (7.14%)	0.66
Hyperlipoidemia	29 (9.60%)	22 (8.46%)	7 (16.67%)	0.094
Coronary atherosclerotic heart disease	12 (3.97%)	10 (3.85%)	2 (4.76%)	0.778
Having two or more abovementioned chronic diseases	53 (17.55%)	47 (18.08%)	6 (14.29%)	0.67
Other chronic diseases	56 (18.54%)	44 (16.92)	12 (28.57%)	0.072

The general demographic characteristics of the subjects are shown in Table 2. Of the 302 patients, 128 (42.38%) were males. There are only 64 people (21.19%) over 65 years old, and most of the interviewees are young and middle-aged people. A small portion (17.55%) of interviewees has multiple chronic diseases. The most of respondents are urban residents (77.81%). In the prior 3 months before the survey, most of the interviewees (59.61%) had taken medication, but very few people (8.94%) had taken more than 3 sorts of medications. There were no significant differences in general demographic and clinical characteristics between people who intended to use the MTM applications and those who did not.

**Table 2:** General demographic characteristics of the subjects.

**Items**	**Total (%) (*n =* 302)**	**Willing to use MTM applications (*n =* 260)**	**Unwilling to use MTM applications (*n =* 42)**	***P*-value**
Male	128 (42.38%)	113 (43.46%)	15 (35.71%)	0.35
Age (>65 years old)	64 (21.19%)	55 (21.15%)	9 (21.43%)	0.97
Educational level (High school or higher)	148 (49.00%)	126 (48.46%)	22 (52.38%)	0.64
Living in urban area	235 (77.81%)	201 (77.31%)	34 (80.95%)	0.59
Having two or more chronic diseases	53 (17.55%)	45 (17.30%)	6 (14.29%)	0.67
>1 h usage of smartphone use per day	159 (52.65%)	138 (53.08%)	21 (50.00%)	0.70
Using medication within past 3 months	180 (59.61%)	155 (59.61%)	25 (59.52%)	0.99
Taking more than 3 sorts of medications within past 3 months	27 (8.94%)	25 (9.62%)	2 (4.76%)	0.31
Good compliance of medication	254 (84.11%)	226 (86.92%)	28 (66.67%)	0.001
Having long-term medication history (≥3 months)	232 (76.82%)	212 (81.53%)	20 (47.62%)	<0.001
Willing to learn about medicine knowledge	281 (93.04%)	249 (95.76%)	32 (76.19%)	<0.001
Using Internet as the main source of medication knowledge currently	75 (24.83%)	70 (26.92%)	5 (11.90%)	0.037
Wanting to get more professional medication knowledge *via* Internet	169 (55.96%)	154 (59.23%)	15 (35.71%)	0.004
Considering medical knowledge on MTM applications too hard to understand	214 (70.86%)	189 (72.69%)	25 (59.52%)	0.081
Worrying user information leakage/disclosure	166 (54.96%)	141 (54.23%)	25 (59.52%)	0.52
Worrying missing reminder information of medication on MTM applications	106 (35.09%)	94 (36.15%)	12 (28.57%)	0.34

Compared with the negative attitude group, a significantly larger percentage of interviewees in the positive attitude group had good compliance with medication (86.92 vs. 66.67%, *P* = 0.001), had long-term medication history (≥3 months) (81.53 vs. 47.62%, *P* < 0.001) and were willing to learn about medicine knowledge (95.76 vs. 76.19%, *P* < 0.001). A larger fraction of interviewees in the positive attitude group were currently using the Internet as the main source to acquire medication knowledge (26.92 vs. 11.9%, *P* = 0.037), and were looking forward to obtaining more professional medication knowledge through the Internet (59.23 vs. 35.71%, *P* = 0.004), compared with negative attitude group.

### Factors Influencing Willingness to Use MTM Applications

To further confirm the relevant influencing factors of interviewees' willingness to use the MTM applications to support chronic clinical practice, we conducted logistic regression analysis. In univariate analysis, we found that the factors most related to intention to use MTM applications were long-term medication history (*P* < 0.001), good compliance with medication regimen (*P* = 0.001), wanting to learn about medication knowledge (*P* < 0.001), using the Internet as the main source of medication knowledge currently (*P* = 0.043) and wanting to get more professional medication knowledge *via* the Internet (*P* = 0.005) (Table 3). Then, the multivariate analysis of the positive attitude group and negative attitude group was carried out with the adjustment of the logistic regression model for 5 variables, and it was revealed that there were distinctly different risk factors from the univariate analyses. As shown in Table 4, in multivariate analysis, the independent risk factors that affected interviewees' willingness to use the MTM applications were long-term medication history (OR = 4.45, [95% CI: 2.07–9.55], *P* < 0.001), willing to learn about medication knowledge (OR = 3.01, [95% CI: 1.05–8.6], *P* = 0.04), and wanting to get more professional medication knowledge *via* Internet (OR = 2.86, [95% CI: 1.36–5.98], *P* = 0.005).

**Table 3:** Univariate analysis of influencing factors associated with the willingness to install and use MTM applications.

**Items**	**Willing to use MTM applications (*n =* 260)**	**Unwilling to use MTM applications (*n =* 42)**	**Univariate analysis**	
			**OR (95% CI)**	***P***
Male	113 (43.46%)	15 (35.71%)		0.35
Age (>65 years old)	55 (21.15%)	9 (21.43%)		0.97
Educational level (High school or higher)	126 (48.46%)	22(52.38%)		0.64
Living in urban area	201 (77.31%)	34 (80.95%)		0.6
Having two or more chronic diseases	47 (18.08%)	6 (14.29%)		0.67
> 1 hour usage of smartphone use per day	138 (53.08%)	21(50.00%)		0.71
Using medication within past 3 months	155 (59.61%)	25 (59.52%)		0.99
Taking more than 3 sorts of medications within past 3 months	27 (8.94%)	25 (9.62%)		0.32
Good compliance of medication	226 (86.92%)	28 (66.67%)	3.32 (1.59–6.94)	0.001
Having long-term medication history (≥3 months)	212 (81.53%)	20 (47.62%)	4.86(2.46–9.61)	<0.001
Willing to learn about medicine knowledge	249 (95.76%)	32 (76.19%)	7.07 (2.79–17.97)	<0.001
Using Internet as the main source of medication knowledge currently	70 (26.92%)	5 (11.90%)	2.73 (1.03–7.22)	0.043
Wanting to get more professional medication knowledge *via* Internet	154 154 (59.23%)	15(35.71%)	2.62(1.33–5.15)	0.005
Considering medical knowledge on MTM applications too hard to understand	189 189 (72.69%)	25(59.52%)		0.084
Worrying user information leakage/disclosure	141 (54.23%)	25 (59.52%)		0.52
Worrying missing reminder information of medication on MTM applications	94 94 (36.15%)	12(28.57%)		0.34

*OR, odds ratio; CI, confidence interval*.

**Table 4:** Multi-variate analysis of influencing factors associated with the willingness to install and use MTM applications.

**Risk factor**	**OR value**	**95% CI**	***P*-value**
Good compliance of medication	0.96	0.32–2.86	0.94
Having long-term medication history(≥3 months)	4.45	2.07–9.55	<0.001
Willing to learn about medicine knowledge	3.01	1.05–8.60	0.04
Using Internet as the main source of medication knowledge currently	2.04	0.65–6.35	0.22
Wanting to get more professional medication knowledge *via* Internet	2.86	1.36–5.98	0.005

*OR, odds ratio; CI, confidence interval*.

### Patients' Future Expectations of Useful Modules of the WeChat Applet of MTM

**Figure 1:**
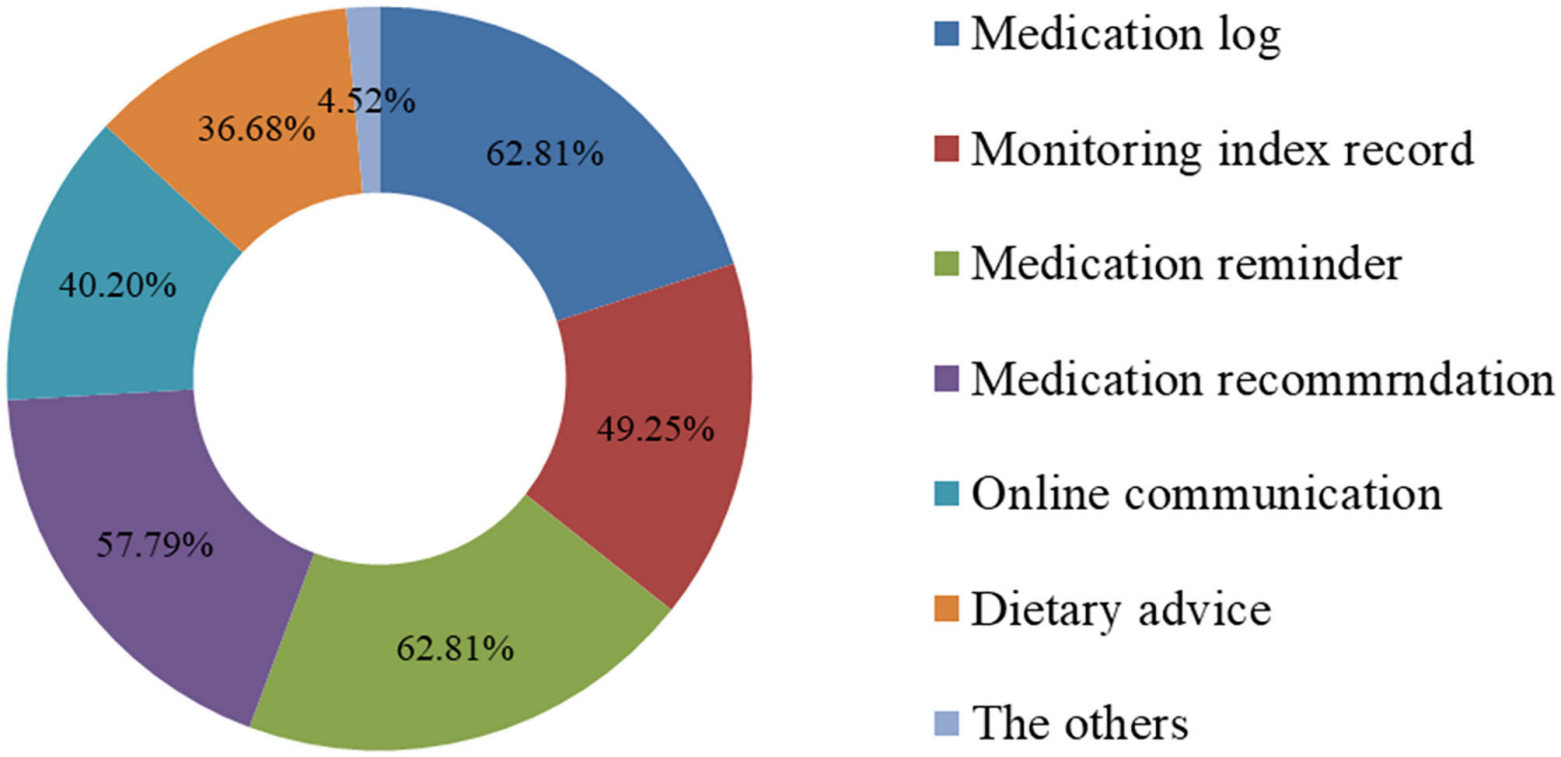
Patients' expectations of functional modules included in the Wechat applet for MTM.

**Figure 2:**
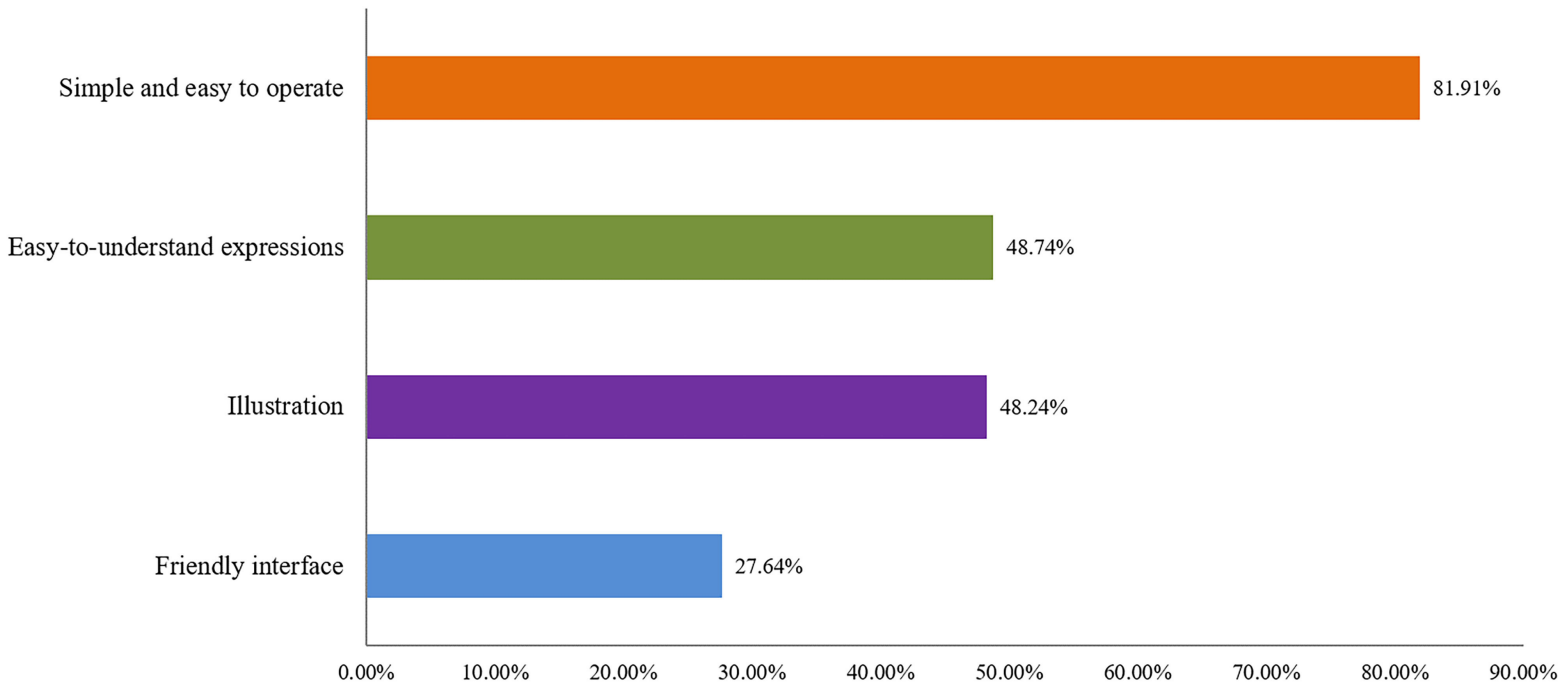
Expectation on the improvement of the Wechat applet for MTM.

We previously found that a total of 260 (86.1%) of the respondents wanted to use the MTM applications. To be more in-depth, the people were more supportive of using the WeChat applet (55%) for chronic disease drug treatment management compared with other MTM applications (23.46%). Other interviewees (21.54%) thought that both forms were acceptable. Through further investigation, it was found that the majority of respondents who accepted the use of WeChat for MTM warranted the future addition of medication log (62.81%), medication reminders (62.81%), and medication recommendations (57.79%) to the WeChat applet (Figure 1). In terms of the appearance of the WeChat applet, as shown in Figure 2, respondents preferred applets with simple and convenient operations (81.91%), followed by applets with illustrations (48.24%), and easy-to-understand expressions (48.72%).

## Discussion

In this project, we created a questionnaire to have knowledge of user needs, preferences, and expectations for MTM applications. We found that the risk factors, including long-term medication history, willingness to learn about medication knowledge, and expectation to get more professional medication knowledge *via* Internet, influenced people's use of the MTM applications for chronic diseases. Further research found that people are more likely to choose the WeChat applet as their desired form of MTM applications.

Our survey results showed that more than half of patients (52.65%) used phones for more than an hour per day, and 86.09% of respondents were willing to use MTM applications, which created a large client market for applications of drug management software. There are many MTM applications on the market, most of which are developed by the software industry, and a few are jointly by healthcare institutes (19). Quite a few studies have focused on the discussion of functional characteristics and pre-effects of the software after it is developed. (23, 24) However, there are very few studies that have investigated the factors influencing users' attitudes toward MTM applications, as well as individual needs that those applications can provide. Emerging evidence has indicated that respondents who have a positive attitude toward receiving MTM are willing to seek MTM medication therapy management services (25, 26). Consistent with our study, we found that the respondents are more likely to use MTM applications for chronic disease treatment if they have a hope to acquire medical knowledge and an interest in more professional medication knowledge online. Additionally, we found that respondents with a long-term medical history were more likely to use MTM applications because they were more aware of their chronic health conditions. Traditional MTM services provided by medical professionals do have their own merits. However, it is difficult for most people to get “face-to-face” advice easily from professionals in the first place because of the distance and cost. With increasingly stronger health awareness and a prospering software industry, patients nowadays become more willing to use corresponding MTM applications (27, 28). In our research, compared with other MTM applications (23.46%), people with chronic diseases are more inclined to use WeChat applets (55.00%) as their desired form of MTM applications. Other interviewees (21.54%) consider that both forms are acceptable. The probable reason why people are more inclined to install WeChat applets for MTM is that only the minority of the mobile phone applications are completely free and most of the applications need to be updated regularly (20), which brings inconvenience to the operation. On the contrary, WeChat applets are integrated into the WeChat platform without downloading, which greatly saves the memory of the phone and makes the program far easier to use. Currently, there are 1.09 billion WeChat users every day, of which 400 million users use the applet (29). The large user base is expected to make the WeChat applet the main tool for medication management. Nevertheless, few studies have reported how the WeChat applet effects medication management. Therefore, we are paying strong attention to respondents' further demands and concerns for WeChat applets. Tabi et al. found that the most prevalent features of mobile apps for MTM are reminders, symptom tracking, and the ability to share data with family members or physicians (19). Similarly, our survey data shows that the majority of patients who are willing to use WeChat applets for chronic MTM service want some useful modules included in WeChat applets, such as medication logs, medicine reminders, and medication recommendations.

As to the people's concerns for the WeChat applet, the majority are worried about the software operation and language expression, which poses an obstacle to the further promotion of WeChat applets. More than 80% of respondents called for a simpler operation of WeChat applets. Nearly half of the respondents hope that the expression of drug knowledge is easy to understand. For example, the professional expression of the usage of acarbose tablets “taking it with meals” should be replaced with “the first mouthful of food and medicine at the same time”.

The study population mainly consisted of patients from health facilities in Hunan Province and most of the patients were urban residents, which may not be representative of the overall condition of patients across China. However, it can still provide some suggestions for future MTM applications to a certain extent that the MTM applications should be improved in several aspects: simpler operation, more illustration, and less professional language expression.

## Conclusion

In this study, we learned through preliminary surveys that the factors affecting the use of MTM applications for patients with chronic diseases, as well as their expectations for the functions and contents of the WeChat applet for chronic disease drug management. This provides some insights for the later development of practical programs to support MTM of chronic disease, especially the WeChat applet in the future.

## Data Availability Statement

The original contributions presented in the study are included in the article/supplementary material, further inquiries can be directed to the corresponding author/s.

## Ethics Statement

The study was approved by Ethics Committee of the First Hospital of Changsha. All respondents gave their informed consent before completing the online questionnaire.

## Author Contributions

SH and JS conceived, designed, and wrote the protocol for this study. JH, XD, and LO were responsible for coordinating the study and the acquisition of the data. GH reviewed the first draft of the manuscript and contributed to the sections of the manuscript. All authors provided a critical review of the manuscript and approved the final version.

## Funding

The research is supported by the scientific research project of the Hunan Health Commission (No. 20200202), the clinical pharmacy research project of Hunan Medical Association (No. HMA202001006), and the Natural Science Foundation of Hunan Province (No. S2022JJKYLH0064).

## Conflict of Interest

The authors declare that the research was conducted in the absence of any commercial or financial relationships that could be construed as a potential conflict of interest.

## Publisher's Note

All claims expressed in this article are solely those of the authors and do not necessarily represent those of their affiliated organizations, or those of the publisher, the editors and the reviewers. Any product that may be evaluated in this article, or claim that may be made by its manufacturer, is not guaranteed or endorsed by the publisher.
